# Monodisperse Sub-100 nm Au Nanoshells for Low-Fluence Deep-Tissue Photoacoustic Imaging

**DOI:** 10.1021/acs.nanolett.3c01696

**Published:** 2023-08-04

**Authors:** Luis D. B. Manuel, Vinoin Devpaul Vincely, Carolyn L. Bayer, Kevin M. McPeak

**Affiliations:** † Gordon and Mary Cain Department of Chemical Engineering, Louisiana State University, Baton Rouge, Louisiana 70803, United States; ‡ Department of Biomedical Engineering, Tulane University, New Orleans, Louisiana 70118, United States

**Keywords:** Au nanoshells, nanoparticles, nanomedicine, photoacoustic imaging, deep-tissue imaging

## Abstract

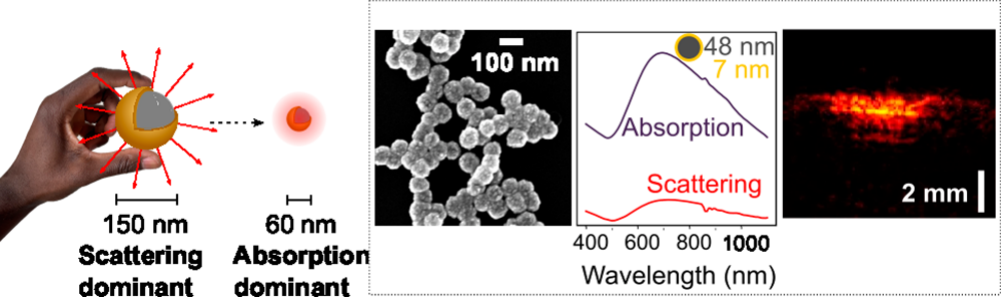

Nanoparticles with high absorption cross sections will advance therapeutic and bioimaging nanomedicine technologies. While Au nanoshells have shown great promise in nanomedicine, state-of-the-art synthesis methods result in scattering-dominant particles, mitigating their efficacy in absorption-based techniques that leverage the photothermal effect, such as photoacoustic (PA) imaging. We introduce a highly reproducible synthesis route to monodisperse sub-100 nm Au nanoshells with an absorption-dominant optical response. Au nanoshells with 48 nm SiO_2_ cores and 7 nm Au shells show a 14-fold increase in their volumetric absorption coefficient compared to commercial Au nanoshells with dimensions commonly used in nanomedicine. PA imaging with Au nanoshell contrast agents showed a 50% improvement in imaging depth for sub-100 nm Au nanoshells compared with the smallest commercially available nanoshells in a turbid phantom. Furthermore, the high PA signal at low fluences, enabled by sub-100 nm nanoshells, will aid the deployment of low-cost, low-fluence light-emitting diodes for PA imaging.

Emerging therapeutic and bioimaging technologies leverage nanoparticles with optical resonances in the near-infrared (NIR) wavelength regime, i.e., the biological window, to efficiently target deep tissue.^1^ This coupling of nanotechnology and biomedicine is commonly referred to as nanomedicine.^2^ Prominent examples include photothermal therapy (PTT),^3^ optical coherence tomography (OCT),^4^ photoacoustic (PA) imaging,^5^ and diffuse optical tomography.^6^ Although these techniques rely on absorption and scattering by endogenous tissue components, exogenous contrast agents can significantly augment signal generation.^2^ Plasmonic nanoparticles, with their strong light–matter interactions and biocompatibility, e.g., Au, make excellent exogenous agents for these applications.^2^ Consequently, Au nanoparticles of different shapes, e.g., nanorods,^7^ nanocages,^8^ bipyramids,^9^ and nanoshells,^10^ are promising candidates for nanomedicine. Among Au nanoparticles of different shapes, spherical particles have the lowest surface-to-volume ratio, which can result in lower toxicity.^11^ Consequently, several foundational studies in nanomedicine use Au spheres, albeit limited to the visible light regime. Au nanoshells are also spherical, support NIR resonances in the biological window, and have been used in pioneering nanomedicine work.^12−14^

Au nanoshells are inorganic structures with a SiO_2_ core covered by a thin Au shell. Nanoshells were developed in the late 90s by the Halas group and rely on coupling of the local surface plasmons resonances between the inner and outer surfaces to offer tunable absorption and scattering in the NIR.^10,15^ There has been significant progress in using nanoshells for nanomedicine applications. Hirsch et al. used nanoshells to destroy cancer cells via PTT in the early 2000s,^16^ and Food and Drug Administration (FDA)-approved clinical trials followed.^17^ Despite their promise for nanomedicine, Au nanoshells can be significantly improved for absorption-based applications.^18,19^ To date, reported studies use large nanoshells that scatter more than they absorb.^12,13,16,18,20−22^ Absorption-dominance in nanoshells requires particles with sub-100 nm diameter given synthetically achievable shell thicknesses.^18,23,24^ The lack of examples in the literature of sub-100 nm nanoshells stems from major synthetic challenges resulting from poor particle stability as the core size decreases.^20^ Additionally, there are physical limitations on how thin the Au shell can be relative to the SiO_2_ core.^20^ As a result, scattering-dominant nanoshells are pervasive in absorption-based nanomedicine. Figure 1a highlights the advantages of moving from large scattering-dominant nanoshells to smaller absorption-dominant nanoshells for absorption-based nanomedicine techniques such as PA imaging.

**Figure 1:**
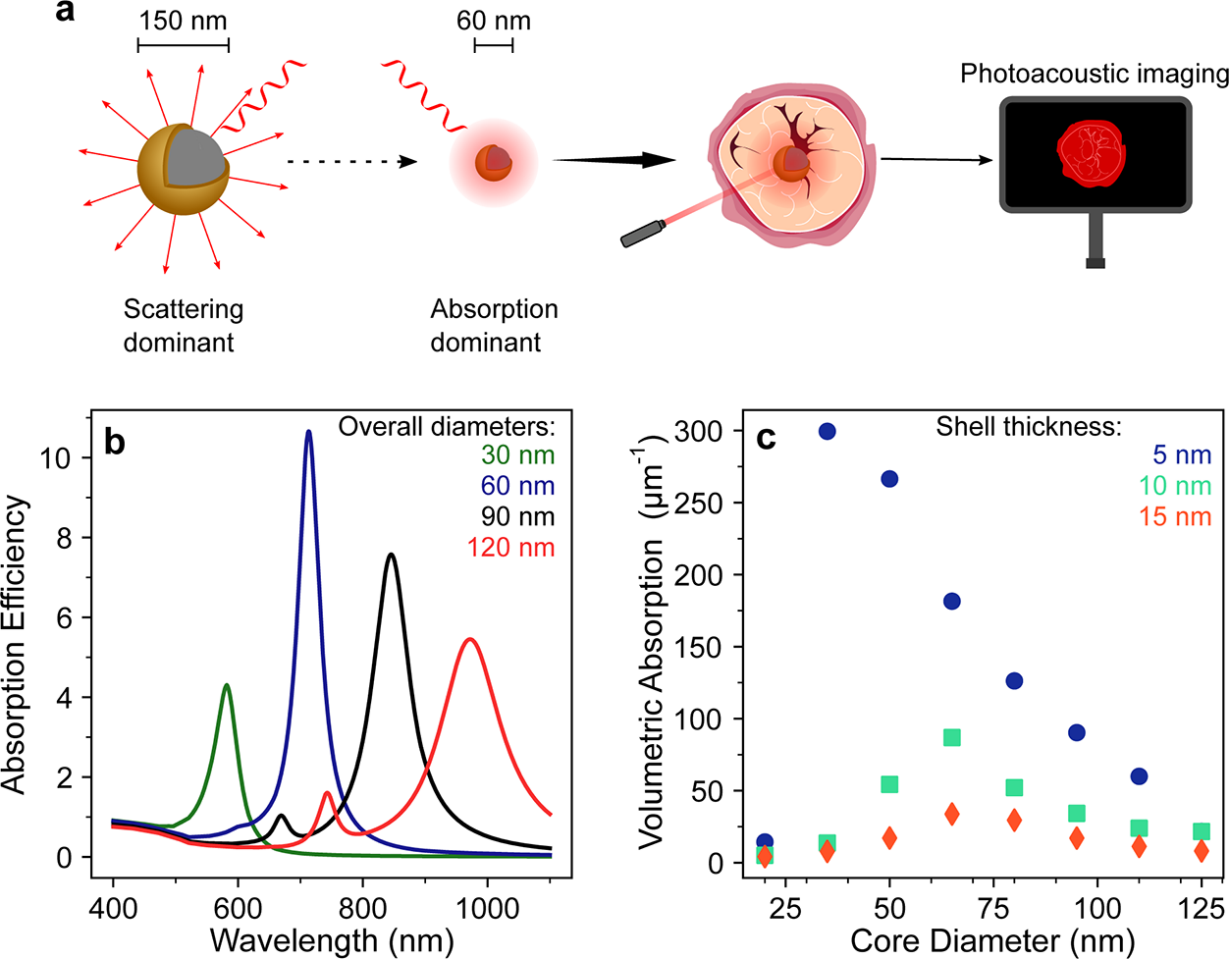
(a) Schematic showing that decreasing nanoshell size leads to absorption dominant optical behavior and improved performance in absorption-based nanomedicine techniques. (b) Simulated absorption efficiency of 5 nm Au shells on 20 (green), 50 (purple), 80 (black), and 110 nm (red) SiO_2_ cores indicates 60 nm overall diameter nanoshells have the highest values in the biological window. (c) Simulated volumetric absorption coefficients of nanoshells of different core diameter with shell thicknesses of 5(blue), 10 (green), and 15 nm (red). The volumetric absorption decreases with both core diameters and shell thickness increase, with nanoshells of sub-100 nm diameters having significantly larger volumetric absorption.

The benefits of synthesizing sub-100 nm nanoshells include improved light absorption and improved transport in tissue.^11^ The improved light absorption can be seen in Figure 1b and Figure 1c, which compare nanoshells of different sizes using Mie theory simulations; Figure 1b shows that nanoshells with an overall diameter of 60 nm, made from a 50 nm core and a 5 nm shell, have higher absorption efficiency than the larger nanoshells commonly seen in the literature. Figure 1c shows that sub-100 nm nanoshells can have significantly higher volumetric absorption than larger diameter nanoshells. Higher volumetric absorption stems from increased absorption efficiency and the number of particles that can fit a given volume as the dimensions decrease. Higher volumetric absorption contributes to more signal generation in absorption-based nanomedicine. Beyond benefiting the absorption properties, reducing the nanoshell size results in improved cellular uptake,^11^ evidenced by studies on spherical gold nanoparticles which show that smaller diameters near 50 nm are better internalized by cells.^25^ Particles that are too small have higher energy requirements, while larger particles diffuse slowly.^11^ Additionally, particles near 50 nm have more optimal clearance pathways and have fewer long-term toxicological implications due to higher clearance rates.^11,26^

Here, we overcome the optical tunability limits of nanoshells and optimize them for absorption-based applications by developing a synthesis method to decrease their overall diameter to less than 100 nm. The smallest nanoshells we synthesized have a 48 nm SiO_2_ core diameter and a 7 nm Au shell thickness, with a total diameter of 62 nm. These nanoshells are absorption dominant and achieve a 14-fold increase in the volumetric absorption coefficient compared to commercial Au nanoshells with dimensions commonly used in nanomedicine. We show the direct implication of their optimized absorption profile by comparing their performance as PA imaging contrast agents with conventional (i.e., >100 nm diameter) nanoshells, and we show that sub-100 nm Au nanoshells have improved performance with a 50% increase in PA imaging depth in a turbid phantom when compared to commercial nanoshells.

The first step in the synthesis is the preparation of SiO_2_ core particles and Au seed particles. The SiO_2_ core particles react with 3-aminopropyltriethoxysilane (APTES) to obtain sites where the Au seeds adsorb in the next step. Before the Au adsorption step, centrifugation cleaning cycles ensure that the nucleation sites for Au shell growth are on the SiO_2_ surface. These cleaning cycles eliminate excess APTES, thus preventing the seeds from attaching to APTES in the solution. After seeding, additional cleaning cycles to remove unbound Au seeds are performed. The final step in the synthesis is the Au shell growth on seeded SiO_2_. We used formaldehyde as the reducing agent in a K_2_CO_3_-aged HAuCl_4_ medium in the presence of NH_4_OH. The inception of NH_4_OH is the major novelty of our process, and it is discussed further below. Attempts to synthesize sub-100 nm nanoshells without the addition of NH_4_OH yielded poor results. Previous attempts at modifying the synthesis process did not allow the consistent synthesis of absorption-dominant sub-100 nm nanoshells.^27−29^

To consistently synthesize sub-100 nm nanoshells, it is necessary to overcome key challenges during the steps listed above. Of these challenges, improving the shell growth step is paramount. Shell growth for sub-100 nm nanoshells is more difficult than conventional large nanoshells due to the decrease in particle stability as the core size decreases.^20^ Sub-100 nm nanoshells also require thinner shells to achieve NIR resonance.^24,30,31^ The core-to-shell ratio dictates resonance wavelength and is highly sensitive to changes in shell thicknesses at small core diameters.^24,30,31^ Therefore, Au shell thickness precision is critical at smaller size regimes. Departure from traditional nanoshell synthesis recipes by adding NH_4_OH during shell growth allowed us to synthesize sub-100 nm nanoshells consistently.

The introduction of NH_4_OH during Au shell growth was inspired by its role as a stabilizer during the Stober synthesis of SiO_2._^32^ The addition of NH_4_OH promotes the formation of hydrogen bonding to the surface of the seeded silica particles through residual THPC ligands on the gold seeds. Consequently, there is an improvement in the hydrophilicity of the particles, leading to improved stability. Figure 2 confirms that this addition does indeed prevent agglomeration. Extinction spectra in Figure 2a, without NH_4_OH, and Figure 2d, with NH_4_OH, show that adding NH_4_OH reduced the full width at half-maximum (fwhm) of the nanoshell extinction spectra. Electron micrographs of drop-cast samples from the two suspensions further support the conclusion that adding NH_4_OH reduces the agglomeration of the nanoshells. Figure 2b,c shows that in the absence of NH_4_OH, significant agglomeration of the nanoshells occurs. The Au shell growth is uncontrollable in the absence of NH_4_OH, with significant Au growth in solution; by contrast, adding NH_4_OH results in improved shell growth and negligible agglomeration, as highlighted in Figure 2e and Figure 2f. The micrographs also indicate that NH_4_OH inhibits the formation of Au in the solution. Adding NH_4_OH raises the pH to >10.1, where Au(OH)_4_^–^ is the major species present.^33^ Out of the possible gold complexes that can be present, Au(OH)_4_^–^ has the lowest redox potential and slowest reaction rate making Au growth in solution less likely.^33^ The Supporting Information provides a complete discussion of the strategies we implemented to address issues at the different steps in the synthesis.

**Figure 2:**
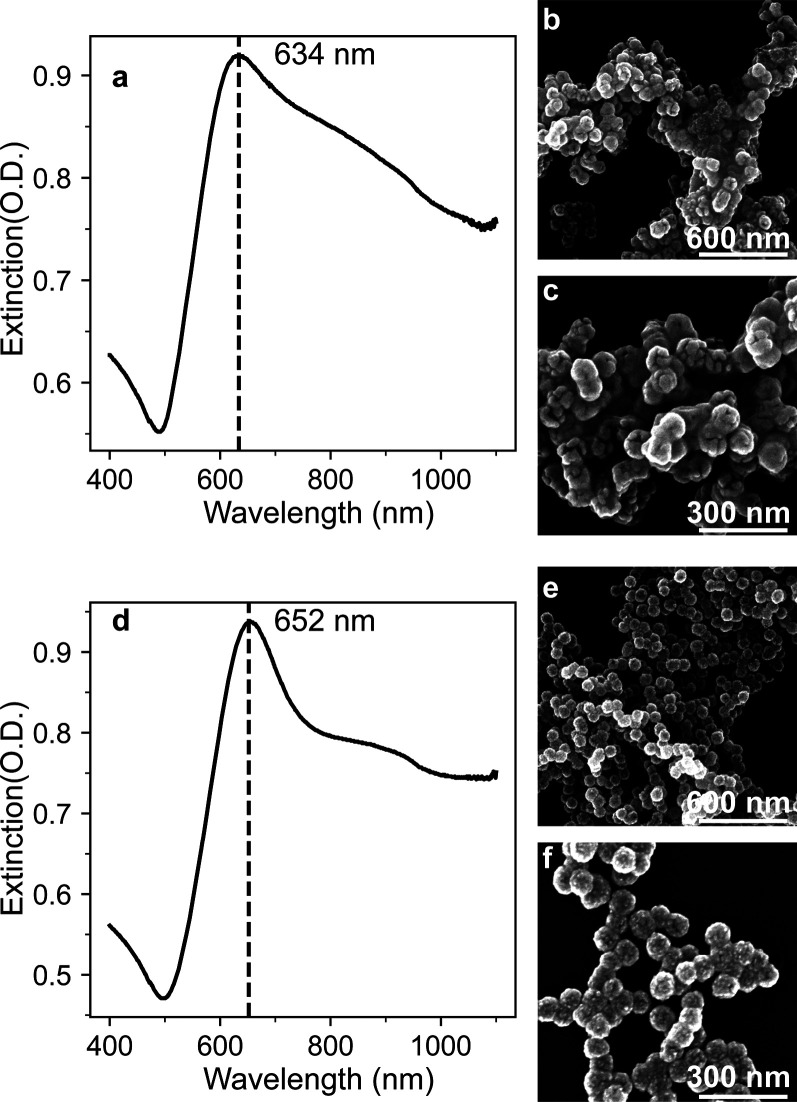
Effect of NH_4_OH addition during Au shell growth. Extinction spectra of Au nanoshell colloids grown (a) without NH_4_OH addition and (d) with 6.88 mM NH_4_OH; electron micrographs of drop cast Au nanoshells (b, c) without NH_4_OH and (e, f) with 6.88 mM NH_4_OH. The addition of NH_4_OH resulted in improved shell growth with narrower peak bandwidth and less aggregation.

Following the above experimental guidelines, we synthesized scattering-dominant and absorption-dominant nanoshells. The electron micrographs in Figure 3a and Figure 3b highlight their size differences. The scattering-dominant nanoshells in Figure 3a have an 80 nm core diameter and an 11 nm shell. In comparison, the absorption-dominant nanoshells in Figure 3b have a 48 nm core and a 7 nm Au shell. We calculated the shell thickness from the difference in the diameters before and after shell growth using electron micrographs and a disk centrifuge photosedimentometer (see Figure S1). The extinction spectra from the two nanoshell dimensions are nearly overlapping; see Figure 3c, which enables us to better compare their absorption and scattering fractions.

**Figure 3:**
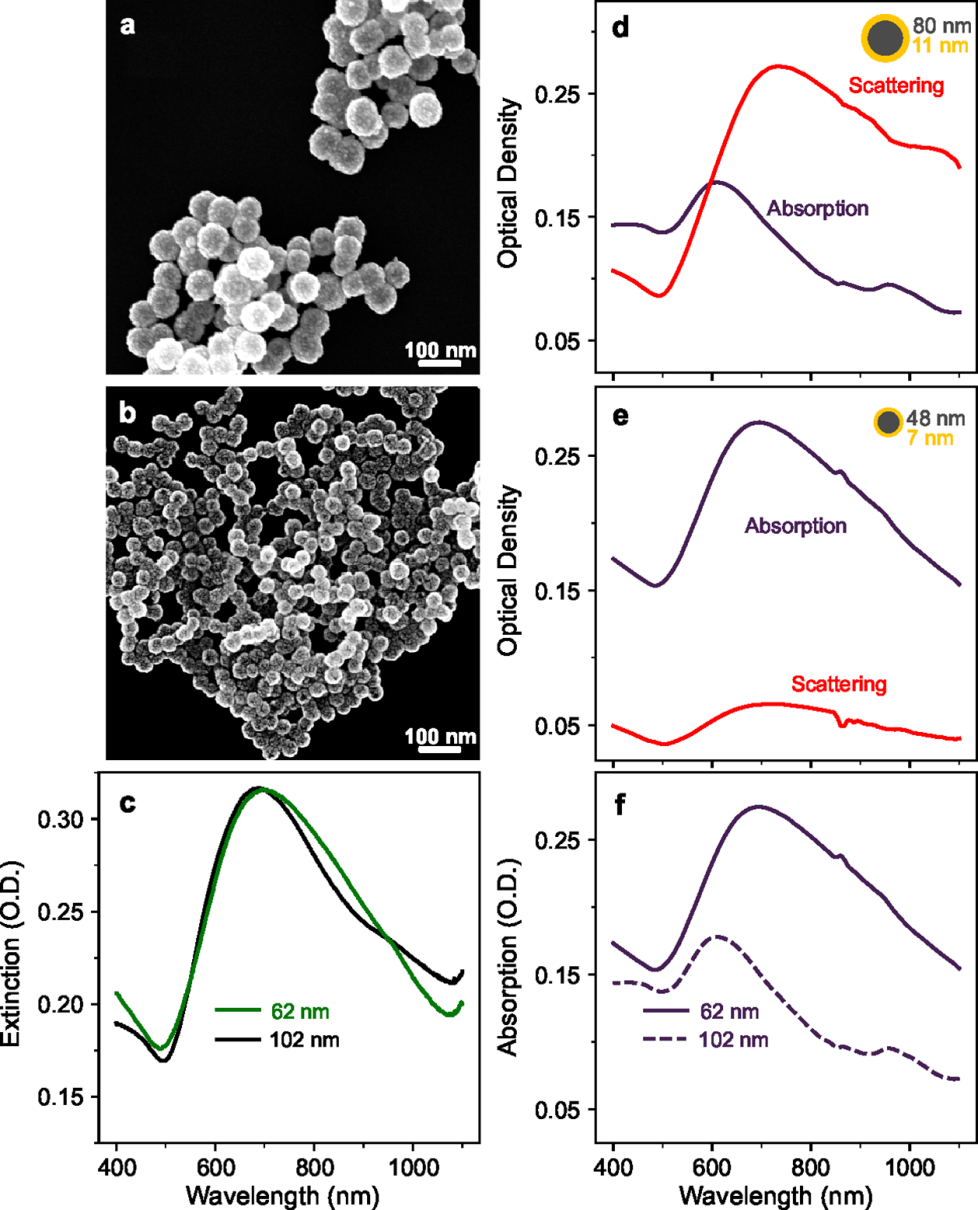
Electron micrographs and optical properties of scattering- and absorption-dominant nanoshells. Electron micrographs of drop-cast nanoshells with outer diameters of (a) 102 nm and (b) 62 nm. (c) Nearly overlapping extinction spectra from suspensions of nanoshells in (a) and (b). (d, e) Absorption and scattering for overall diameters: (d) 102 nm scattering dominant nanoshells, (e) 62 nm absorption dominant nanoshells; (f) absorption comparison for 102 nm vs 62 nm nanoshells, where 62 nm nanoshells have higher and more redshifted absorption.

To experimentally show how the nanoshell dimensions affect their optical behavior, Figure 3d,e shows the spectra of the nanoshells shown in the micrographs. The larger nanoshells are confirmed to be scattering, while the smaller nanoshells are absorption dominant. Typical spectrophotometric measurements on colloidal samples measure extinction only via light transmission to the detector through the sample. Extinction is the summation of absorbed and scattered light. We separate the nanoshell absorption and scattering effects using an integrating sphere with a center-mounted cuvette, enabling the detector to collect light from all directions. Our method involves a two-step measurement (see Supporting Information Figure S4). In the first step, we allow transmitted and scattered light to reach the detector. In the second step, a light trap opposite the entrance of the integrating sphere prevents transmitted light from reaching the detector. The second measurement provides the scattered fraction. The difference between the two measurements determines the transmitted fraction. We then calculate the absorbed fraction using Kirchoff‘s rule, which states that the absorbed, scattered, and transmitted fractions sum to 1.

As predicted by Mie’s theory in Figure 1b, smaller nanoshells absorb light more efficiently than larger nanoshells. Table 1 shows the measured maximum volumetric absorption in the NIR of four nanoshells with varying dimensions: the two synthesized nanoshells shown above, and two commercial nanoshells. The commercial nanoshell with a 118 nm core and 15 nm shell represents the most commonly used dimensions in absorption-based nanomedicine reports,^12,13,15,16^ while 81 nm core diameter and 20 nm shell were the smallest commercially available nanoshells. According to Bohren and Huffman, volumetric absorption, defined as normalized absorption cross section per particle volume, is the most practical way to measure efficiency toward applications.^30,34^ From Table 1, the 62 nm absorption dominant nanoshells have the highest volumetric absorption, which is 14-fold larger than the volumetric absorption of the nanoshells used in the literature.

**Table 1:** Measured Maximum Volumetric Absorption of Two Synthesized Nanoshells Compared to Two Larger Commercial Nanoshells

core diameter (nm)	shell thickness (nm)	total diameter (nm)	λ_abs_^a^ (nm)	*V*_abs_^b^ (μm^–1^)
48 ± 5	7	62 ± 5	686	304.08
80 ± 7	11	102 ± 7	676	67.49
81 ± 8	20	125 ± 9	650	23.54
118 ± 4	15	147 ± 7	823	21.80

a λ_abs_ represents the maximum NIR absorption wavelength.

b *V*_abs_ represents the maximum volumetric absorption.

To better understand the role of scattering and absorption in nanomedicine-enabled bioimaging modalities, we tested absorption-dominant and scattering-dominant nanoshells as exogenous contrast agents in PA imaging. Motivated by prior work using plasmonic nanoparticles as contrast agents for PA imaging, we chose PA as the model application.^35^ PA imaging employs the absorption of nanosecond pulsed light to generate ultrasound waves via the thermoelastic effect.^36^ PA signal generation is governed by the equation below:
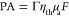
1where Γ is the Grüneisen parameter, η_th_ is the thermal conversion efficiency, μ_a_ is the absorption coefficient, and *F* is the local fluence.

A comparison between absorption-dominant and scattering dominant nanoshells was conducted at the same extinction optical density of 1 to better elucidate the role of light absorption. Note that the optical density was confirmed using a UV/vis/NIR spectrometer and prior to imaging with an in-house plate reader.

The photostability of the absorption-dominant particles was first assessed, and the PA signal was linear up to fluence values of 125 mJ/cm^2^, with a lack of hysteresis in the signal as the fluence returned to 40 mJ/cm^2^ (Figure S7). We measured the PA imaging depth for the different Au nanoshells by placing the nanoshell dispersions in a tube placed diagonally under a turbid phantom, e.g., 1 wt % aqueous dispersion of 1 μm diameter polystyrene spheres. The sketch in Figure 4a details the experimental setup for the PA depth measurements. The diagonal orientation of the tube allows the optical path to be varied by adjusting the imaging plane (IP) along the length of the tube. Parts b–e of Figure 4 are the PA images collected from the tube carrying the 62 nm (parts b and d) and 102 nm (parts c and e) nanoshells at depths of 3 cm (parts d and e) and 6 cm (parts b and c), respectively. Figure 4f plots the PA signal generated by the Au nanoshells at different depths within the turbid phantom. The mean PA signal generated by the absorption-dominant nanoshells outperforms the scattering dominant nanoshells at all imaging depths. PA signals generated at a depth of 4.5 cm from the absorption-dominant particles are similar to those generated by the scattering particles at a shallower depth of 3 cm. This 50% increase in imaging depth motivates the use of the absorbing particles for deep tissue imaging and their ability to generate higher PA signals at lower fluence values.

**Figure 4:**
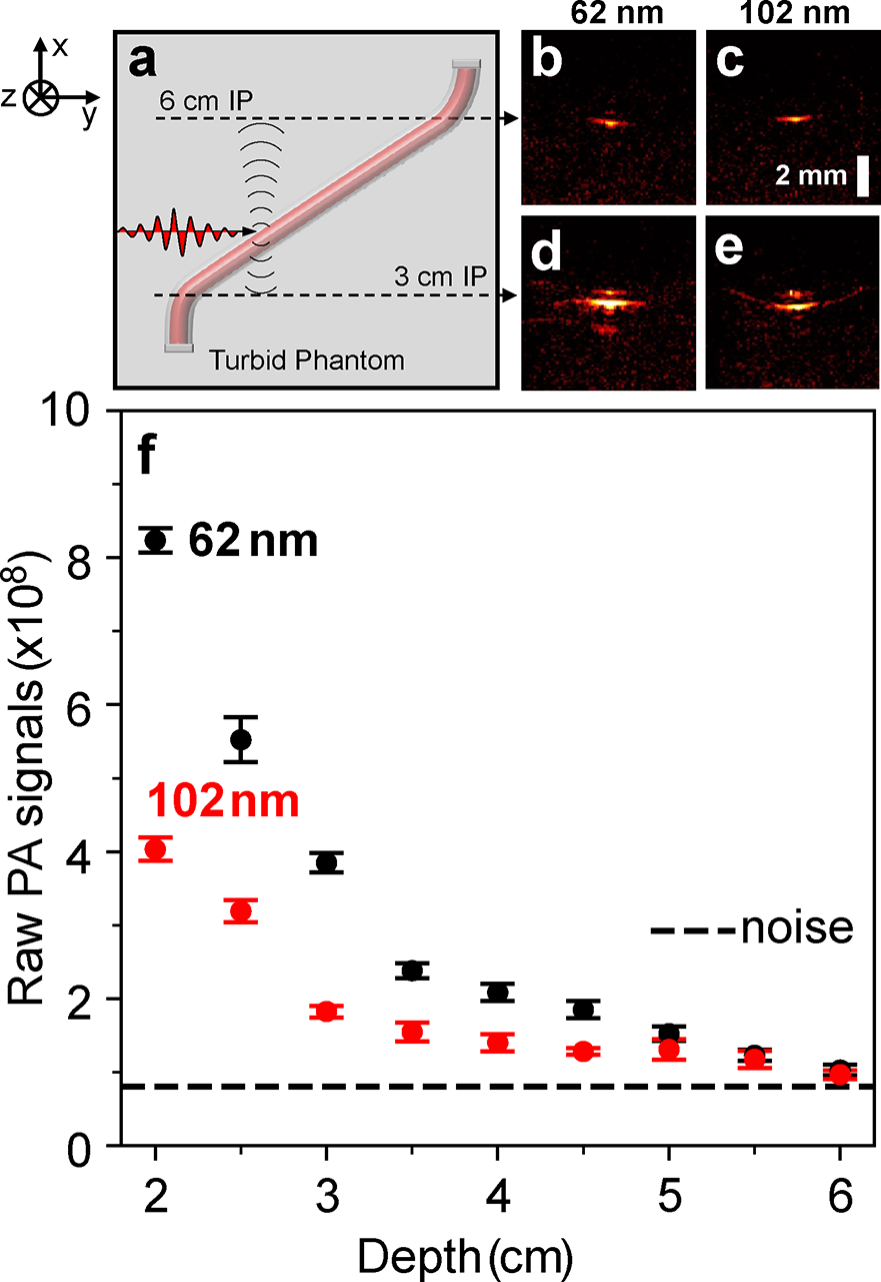
Exploring maximum imaging depth with Au nanoshells in a turbid phantom of suspended polystyrene spheres (PS). A tube carrying the Au nanoshells was placed diagonally under a turbid phantom. This allows the optical path to be varied by adjusting the imaging plane (IP) along the length of the tube. (b)–(e) describe the PA images collected from the tube carrying the 62 nm (b, d) and 102 nm (c, e) nanoshells at depths of 3 cm (d, e) and 6 cm (b, c), respectively. (f) Plot of PA image signal generated by the Au nanoshells at different depths within the turbid phantom. PA signals generated by the 62 nm at a depth of 4.5 cm are similar to those generated at a depth of 3 cm by the 102 nm Au nanoshells.

Improved PA imaging performance at low fluences has several merits. Laser fluence decreases significantly as tissue depth increases.^37,38^ For example, Raijan et al. report a 4-order magnitude change in fluence at 3 cm tissue depth.^39^ In a scenario where fluence values drop significantly, absorption dominant nanoshells can enhance contrast. Additionally, PA systems are moving toward light-emitting diodes (LED) and pulse laser diodes (PLD) as inexpensive alternatives to commonly used solid-state light sources.^40−42^ These systems can translate better to clinical applications since they are less bulky and affordable.^43^ However, PLD and LED light sources operate at lower fluence values, which can result in poor PA image quality.^44^ Absorption-dominant nanoshells can prove beneficial in such applications.

Figure 5a–c compares the photoacoustic performance of our sub-100 nm nanoshells to the smallest commercially available nanoshells (NANOCOMPOSIX) at low fluences of 2 mJ/cm^2^, again restricting the comparison to nanoshells with similar extinction peaks. Figure 5d–f also shows the respective sizes, absorption, and scattering spectra for the various nanoshells. Here we highlight the trend of photoacoustic signals at low fluences, showing a positive correlation between photoacoustic signals and absorption. Figure 5a–c shows a gradual increase in the photoacoustic signal as the corresponding absorption shown in Figure 5d–f increases. The commercial nanoshells of 81 nm core and 20 nm shells have the lowest photoacoustic signals and absorb the least light; see Figure 5a,d. The poor image quality in Figure 5a results from a low contrast-to-noise ratio attributed to a negligible PA signal from 2 mJ/cm^2^, the minimum fluence that PA images could be obtained using commercial nanoshells. On the other hand, absorption-dominant nanoshells show a significantly higher PA signal (Figure 5c) and consequently 50% improvement in PA imaging depth over the smallest commercial Au nanoshell demonstrated in Figure 4f.

**Figure 5:**
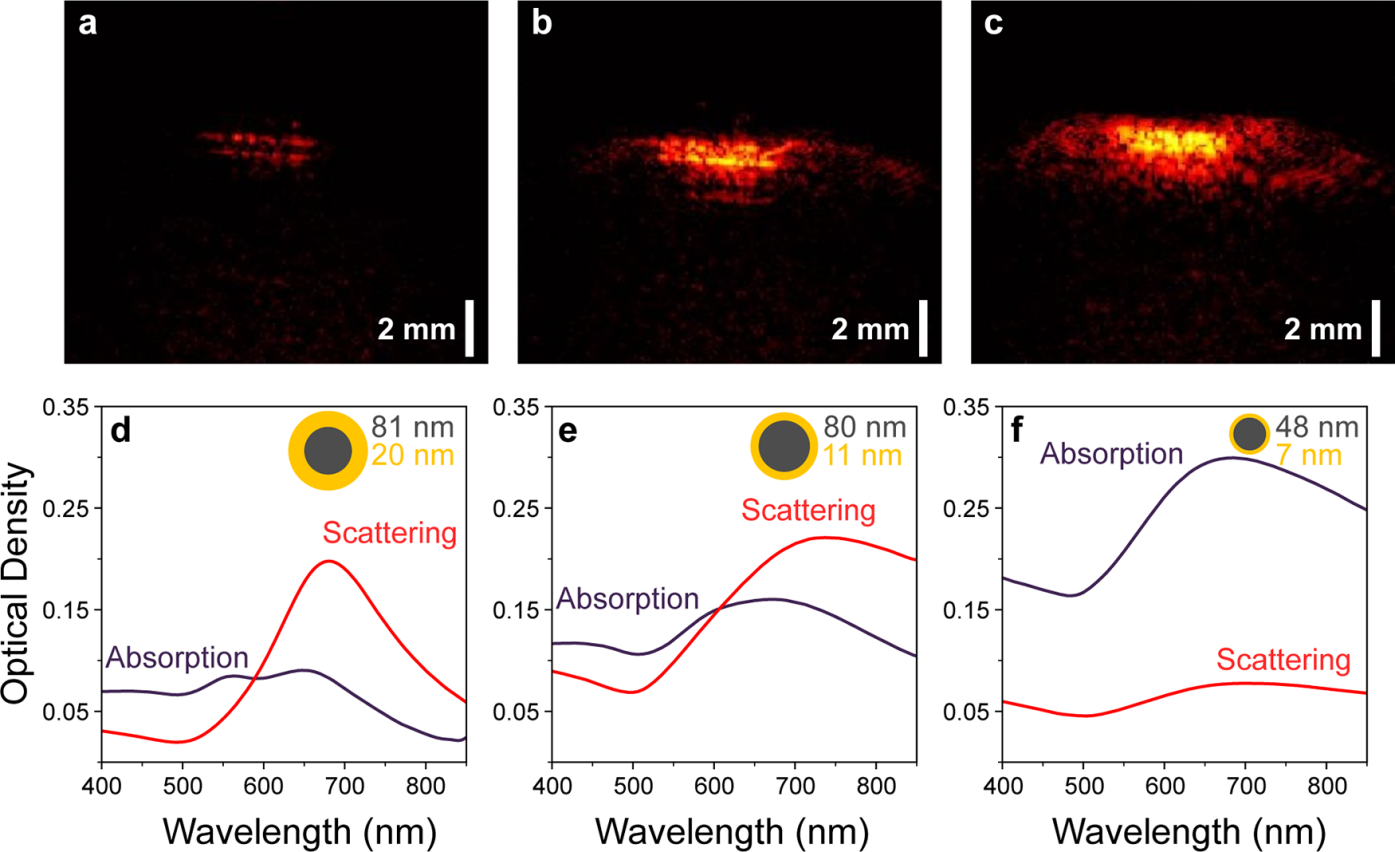
Comparison of low fluence photoacoustic performance and optical properties for sub-100 nm synthesized nanoshells to the smallest available commercial nanoshells. (a–c) PA images of the highest signals at 2 mJ/cm^2^ for (a) commercial nanoshells, (b) scattering-nanoshells, and (c) sub-100 nm absorption-nanoshells. (d–f) Absorption and scattering spectra of nanoshells, with dimensions shown in the inset: (d) commercial, 81 nm core and 20 nm shell; (e) scattering-dominant nanoshells, 80 nm core and 11 nm shell; (f) absorption-dominant nanoshells, 48 nm core and 7 nm shell.

In summary, we have shown how to overcome the current limitations of Au nanoshells, addressing issues at all stages of the synthesis process and leading to the successful realization of absorption-dominant sub-100 nm nanoshells. The synthesized sub-100 nm nanoshells resulted in a 14-fold increase in volumetric absorption coefficient compared to commercial Au nanoshells with dimensions commonly used in absorption-based nanomedicine. Furthermore, we demonstrated the benefits of absorption dominant Au nanoshells on PA imaging by testing sub-100 nm nanoshells and conventional scattering-dominant nanoshells. Our results showed that absorption-dominant sub-100 nm nanoshells outperform conventional scattering nanoshells at low fluences. Consequently, sub-100 nm nanoshells can yield a 50% increase in PA imaging depth in turbid phantoms, as compared to the smallest commercially available nanoshell, and facilitate the use of low-cost PA light sources. Similar studies could be extended to other absorption-based nanomedicine applications that rely on the photothermal effect.

## Supporting Information Available

Materials, nanoshell synthesis procedure and challenges, size analysis results, and optical and photoacoustic characterization methods including setup schemes (PDF)
