# Supplementary material for: Monodisperse Sub-100 nm Au Nanoshells for Low-Fluence Deep-Tissue Photoacoustic Imaging
Source: Nano Lett. 2023 Aug 4;23(16):7334–40. doi: 10.1021/acs.nanolett.3c01696 (PMC10450810; doi:10.1021/acs.nanolett.3c01696)
Supplement: Supplementary file 1 — nl3c01696_si_001.pdf [file nl3c01696_si_001.pdf]

Supporting Information for:

Monodisperse Sub-100 nm Au Nanoshells for Low-Fluence Deep-Tissue Photoacoustic Imaging

*Luis D. B. Manuel[1], Vinoin Devpaul Vincely[2], Carolyn L. Bayer[2], Kevin M. McPeak[1]**

[1]Gordon and Mary Cain Department of Chemical Engineering, Louisiana State University, Baton Rouge, LA 70803, USA. [2] Department of Biomedical Engineering, Tulane University, New Orleans, LA, 70118, USA.

Corresponding Author E-mail*: kmcpeak@lsu.edu

Contents:

1. Materials 2
2. Nanoshell synthesis 2
a. Recipe 2
b. Challenges 3
3. Size Analysis 6
4. Optical Properties 9
5. Photoacoustic Imaging 11

1. Materials

Tetraethoxysilane (TEOS), Tetrakis(hydroxymethyl)phosphonium chloride (THPC), Potassium Carbonate (K2CO3), and 37 % Formaldehyde were purchased from Fischer Scientific, Ethanol, Sodium Hydroxide, Sodium Chloride, and 30 % Ammonium Hydroxide were purchased from VWR, 3-Aminopropyltriethoxysilane was purchased from Acros Organics, Hydrogentetrachloroaurate (III) (HAuCl4) was purchased from Alfa Aesar.

2. Nanoshell synthesis

a. Recipe

The synthesis procedure was modified from Oldenburg et al., briefly:[1] SiO_2 core particles were synthesized from the Stober method where 2.5 ml for 50 nm (1.87 ml for 80 nm) 28% NH_4OH in H2O is added to 35 ml (25 mL for 80 nm) Ethanol while stirring, after 10 min, a mixture of 1 ml Tetraethoxyosilane (TEOS) and 5 ml Ethanol is added dropwise, the reaction proceeds for 24 hrs. The final TEOS concentration is slightly higher for 80 nm cores compared to 50 nm, while all other concentrations remain unchanged. Note that it is critical to perform this synthesis in plastic containers, as most Silanes tend to bind to glass. Au seeds were synthesized following the procedure from Duff et al.,[2] where 1 mL 1M NaOH, 1.5 mL, 1 mL Tetrakis(hydroxymethyl)phosphonium chloride (THPC), and 28 mM $HAuCl_4$ are added sequentially to 30 mL of Deionized H_2O stirred in a strong vortex, allowing 5 minutes between each addition. Note that the $HAuCl_4$ is injected quickly. Synthesized SiO_2 reacts with 3-Aminopropyltriethoxysilane (APTES) overnight at room temperature followed by 1h at 80 °C. The APTES-SiO_2 is then centrifuged washed, and the reaction is repeated; followed by 7

centrifuge wash cycles at 1750 RCF for 110 min for 50 nm SiO_2 core and 55 min for 80 nm SiO_2 core. NaCl and APTES functionalized SiO_2 are added to the 2nm Au colloid, and the seeding reaction proceeds for 16h. The Au-seeded SiO_2 is cleaned via 3 centrifuge wash cycles at 850 RCF for 30 min for 50 nm SiO_2 core and 15 min for 80 nm SiO_2 core. Au shell is grown by adding 50 uL Au-seeded SiO_2 $3x10^{10}$ particles/ml to 500 uL K_2CO_3 aged $HAuCl_4$, followed by adding 5 uL 6.88mM NH_4OH and 10 uL Formaldehyde. The Au-seeded SiO_2 concentrations used for the shell growth step are $3x10^{10}$ particles/ml for 50 nm cores and $1.45x10^{10}$ particles/ml.

CAUTION, $HAuCl_4$ in the presence of NH_3 can form fulminating gold, which is explosive.[3]Take extreme care when mixing $HAuCl_4$ and NH_4OH. Use a dilute amount of NH_4OH, less than a 3 molar ratio to $HAuCl_4$, and do not dry the reaction mixture.[3]

b. Challenges

Stober synthesis is a common approach to synthesizing nearly monodisperse SiO_2 colloidal particles. However, it is challenging to synthesize SiO_2 particles of 50 nm diameter near the lower limits of the Stober process.[4] The synthesis at this lower size limit often leads to particles with irregular shapes and large polydispersity. This large polydispersity can lead to a broadening of the spectral bandwidth. Furthermore, the final particle size is sensitive to the concentration of all reagents, and different reagent combinations can yield similar-sized SiO_2 spheres. TEOS and NH_4OH play more crucial roles than other reagents. Tetraethoxysilane concentration and quality will influence the polydispersity of the nanoparticles. Low concentrations of NH_4OH will result in smaller-sized particles. However, there is a lower concentration limit since NH_4OH stabilizes SiO_2 during the synthesis. Controlling the temperature of the reaction is also critical to the synthesis. Increasing the reaction temperature leads to a larger number of nuclei formed, which also lowers the polydispersity. Optimizing the reaction temperature, and the TEOS to NH_4OH relative

concentrations allows for the consistent synthesis of SiO_2 particles in the lower limits of the Stober process.[5]

A major challenge throughout the synthesis procedure is the propensity for aggregation during different steps. The removal of unbound Aminosilane via centrifugation ensures that Au seeds only bind to the SiO_2 surface. Similarly, it is necessary to remove unbound Au seeds post-seeding to minimize the likelihood of Au growth in the solution from unbound seeds. However, centrifuging at high forces can cause the particles to agglomerate. High rotational centrifugal forces on weakly stabilized nanoparticles will bind them together irreversibly. Small nanoparticles are weakly stabilized due to low charge density. Aggregation can also occur during Au seeding. There is a need to prevent electrostatic repulsion of the seeds on the SiO_2 surface to increase the seeding density. Higher seeding density leads to more continuous shells. One approach for higher seeding density involves adding counter ions such as NaCl to the seed colloid to shorten the debye length of Au particles. This addition reduces the stability. The reduction in stability will make the seeded SiO_2 prone to aggregation. Consequently, the lower stability promotes aggregation during shell growth. Additionally, it affects the post-seeding centrifugation cycle time and the shelf life of the seeded SiO_2.

The shell growth step also has additional challenges. Aging the $HAuCl_4$ growth solution in the presence of K_2CO_3 shifts the pH and ensures that the reaction only takes place on the SiO_2 surface. However, the aging restricts the shell growth between 24 and 72 hours post K_2CO_3 /$HAuCl_4$ solution preparation for optimal results. Additionally, the difficulty of growing Au shells increases as thickness decreases, and thinner shells can be discontinuous. The size of the Au seed particles will limit the minimum thickness of the shell. Thin Au shells are necessary for NIR resonance on small cores because the core-shell ratio dictates resonance wavelength. Small changes in Au

thickness cause significant changes to the core-to-shell ratio of the small-diameter core. Therefore, Au shell thickness precision is very critical at smaller size regimes.

Low rotational centrifugal forces and increased centrifuging times are necessary to overcome the aggregation promoted by weak electrostatic stability. Additionally, it is necessary to improve shell growth via the introduction of NH_4OH, as discussed in the main text. **Table S1** below summarize the challenges and how to address them:

Table 1. Challenges and solutions in ultra small nanoshell synthesis

Problems	SiO_2 cores solutions	Gold seeding solutions	Shell Growth solutions
Polydisperse shapes	• Optimizing TEOS/NH_4OH concentrations and temperature<br>• Dropwise addition of TEOS mixed in ethanol	• Seeding time between 12h and 18h<br>• Low SiO_2 core to Au seed colloid ratio for minimum ethanol addition	• Addition of NH_4OH<br>• Ultrasonication before the addition of formaldehyde
Particle aggregation	• Removal of unattached APTES from SiO_2 colloid<br>• Low rotational centrifugal forces for long times post APTES reaction	• Removal of unbound seeds<br>• Low rotational centrifugal force for long times post seeding	• Removal of unreacted Formaldehyde<br>• Post growth pegylation<br>• Storing in the refrigerator
Incomplete shell	• Reacting cores with APTES twice for high-site density<br>• 2 hours of heating APTES functionalized cores to strengthen covalent bonds	• Excess addition of Au seeds for high seeding density<br>• Decrease debye via length via NaCl addition for high seeding density	• Optimum Kgold age between 48 and 72 hrs<br>• Addition of NH_4OH

3. Size Analysis

Disk Centrifuge Photosedimentometry (DCP) and Scanning Electron Micrography (SEM) was employed for size analysis. DCP is a technique that provides fast and accurate size distribution of a colloidal sample. This technique relies on particle settling time to its diameter and density. In this technique, particles settle through a fluid with a density gradient inside a disk centrifuge. The particle settling time is used to determine diameter and polydispersity from Stoke's law. We used a CPSinstruments disk centrifuge for particle size analysis operating at 24000 RPM, room temperature, using an 8% to 24% Sucrose in a water density gradient. The density gradient is made from 9 solutions of 1.6 ml volume, each from a different ratio of 8 % Sucrose to 24 %. The first solution has 1.6 ml of 8% Sucrose while the last has 1.6 ml of 24 % sucrose. There is a series of solutions in which the amount of 8 % Sucrose by .2 ml while increasing the amount to 24 %. This series is added to the instrument from highest concentration to lowest once the centrifuge reaches the maximum speed of 24000 RPM. A potential source of error include loss of density gradient stability over time resulting in skewed data.

We collected SEM micrographs using a Hitachi S4500 at 20 kV accelerating voltage and 7 mm working distance. ImageJ was used to measure the overall diameters of at least 200 particles in the SEM micrographs.

Additionally, dynamic light scattering (DLS) measurements were conducted using a Malvern Zetasizer Nano ZS

The size analysis results are shown below in Figures S1-S3:

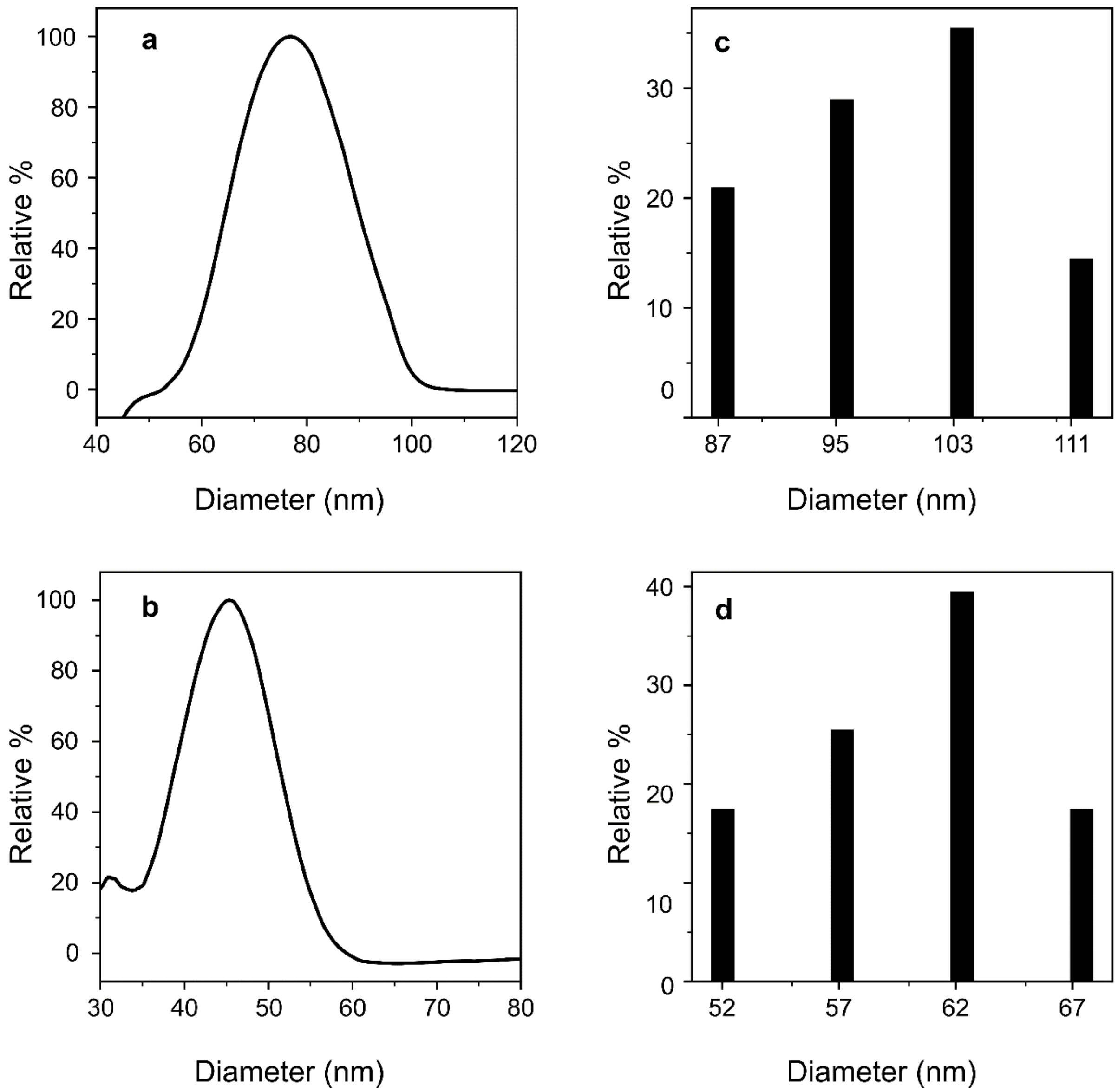

Figure S1. Size analysis results using disk centrifuge photosedimentometery on SiO_2 cores with mean diameters and coefficients of variance: a) 80 nm and 11 % b) 48 nm and 10 % ;c,d)Nanoshell histograms with mean diameter and coefficients of variance c)102 nm and 7 % d)62 nm and 2 %. Number of bins determined from Sturges'rule on at least 200 particles

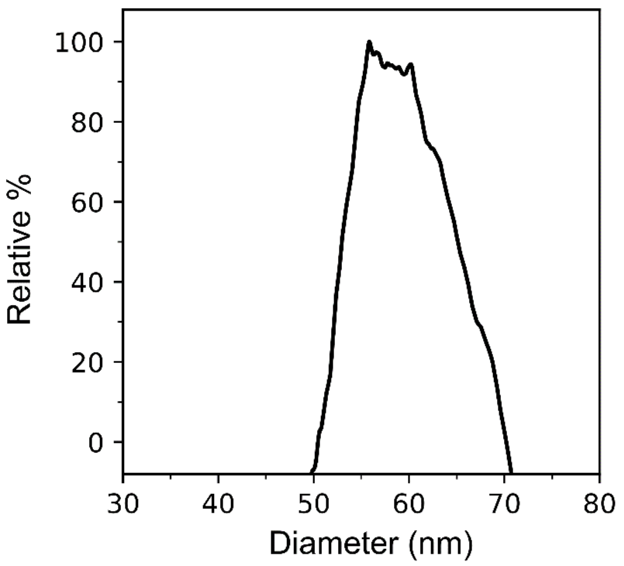

Figure S2: Size analysis results using disk centrifuge photosedimentometery on 62 nm nanoshells indicating 62 nm average diameter and 12.22 % coefficient of variance

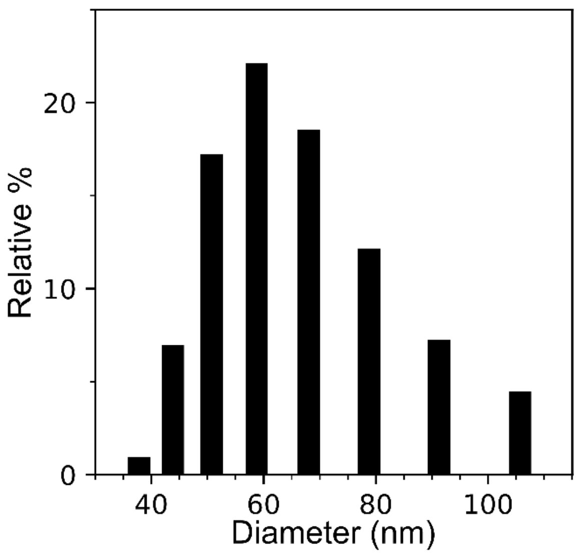

Figure S3: Size analysis results using DLS on 62 nm nanoshells indicating 74 nm average Hydrodynamic diameter and 34 nm standard deviation and PDI value of 0.174

4. Optical Properties

We measured absorption and scattering by center-mounting a cuvette in PerkinElmer Lambda 900 with a 150 mm integrating sphere in two measurements; in the first measurement, a reflection port is placed opposite to the light entering the integrating sphere; for the second measurement, a light trapped replaces the reflection port. Additionally, we collected extinction measurements using a Beckman Coulter DU 730 UV-VIS Spectrophotometer.

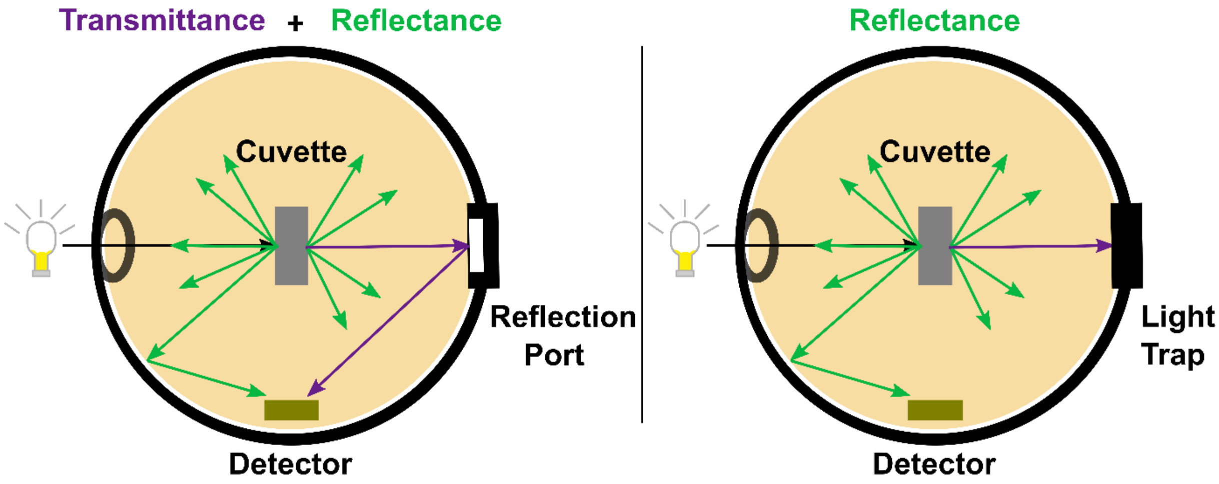

Figure S4. Two-step measurement procedure to separate extinction components: the first step collects transmitted and scattered light, and the second step collects scattered light only. All components can be determined since absorbed, scattered, and transmitted fractions sum to 1

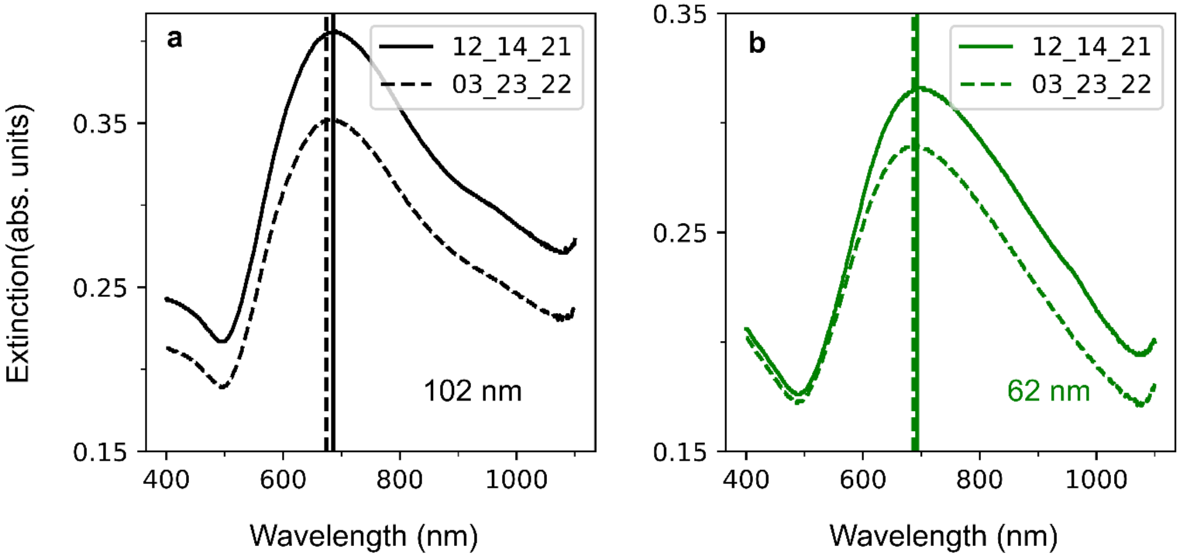

Figure S5: Extinction comparison of 3 months on nanoshells with overall diameter a) 102 nm; b) 62 nm

Variation of absorption coefficient with fluence is shown below:[6]

$$\mu_a = \sigma_a \frac{N_0}{1+\frac{F}{\tau I_{sat}}} \qquad (1)$$

where σ_a is the absorption cross-section, N_0 is the population density, τ is the laser pulse duration, and I_{sat} is the saturation intensity and F the Fluence.

Additionally, absorption cross-section can be calculated from the measured absorption coefficient using Avogrado's number N_a as follows:[7]

$$\sigma_a = \frac{0.2303\times\mu_a}{N_a} \qquad (2)$$

Note that the absorption coefficient is determined from the measured spectra normalized by the pathlength (1 cm) and the final nanoshell concentration. Final nanoshell concentration was calculated from the Au mass present determined using ICP-OES after Aqua Regia digestion using a PerkinElmer Optima 8000 Optical Emission Spectrometer (OES).

5. Photoacoustic Imaging

Optical excitation was produced using a pulsed optical parametric oscillator (OPO) laser (Phocus BENCHTOP, Opotek Inc., Carlsbad, CA, USA) operating at 700 nm wavelength with a 10 Hz repetition rate and a 5 ns pulse width. The pulse-to-pulse variation was maintained under 5%. The light was focused using a converging lens to an illumination area of 0.5 ± 0.1 mm and a fluence of 15 mJ/cm^2 on the surface of the imaged phantom. The optical energy density incident on the phantom surface was measured using a power meter (Ophir Technologies, West North Logan, UT, USA). The surface fluence was calculated by dividing the measured average energy by the illumination area. The laser was externally triggered using a function generator via a custom integration with an open-architecture data acquisition system (Vantage 256, Verasonics Inc., Kirkland, WA, USA) that was coupled to a 6 MHz linear array transducer (L7-4, Philips, Amsterdam, Netherlands). A front-end preamplifier (Legion Amplifier, Photosound Inc., Huston, TX, USA) was used to increase the sensitivity to low frequency signals, providing a dynamic range of ~85 db. The photoacoustic signal acquired by the system was linear within the laser energy range tested (R^2 = 0.95). The transducer was positioned such that the imaging plane was perpendicular to the direction of optical illumination (Figure S6a). The imaging phantom for testing the maximum PA imaging depth with the nanoshells was a 1% dispersed suspension of 1-micron polystyrene sphere (07310-15, Polysciences Inc., Warrington, PA 18976, USA) with a

vinyl tube (BB31785-V/10, Scientific Commodities Inc., Lake Havasu City, AZ) carrying the synthesized nanoshells placed diagonally in the box. The tubing has an internal and outer diameter of 1.57 mm and 2.08 mm, respectively. This diagonal orientation of the tube allows for imaging at varying depths (2-6 cm) from the surface of illumination, by focusing the transducer along the length of the tube. Using Mie Theory, the reduced scattering of the dispersed suspension was estimated to be a value of 0.75 cm^{-1} at 700 nm.[8] PA imaging was sequentially performed from a depth of 2-6.5 cm at 0.5 increments for the 62 nm & 102 nm shells. Image acquisition was controlled through a MATLAB 2022a (Mathworks Inc., CA, USA) graphical interface, and images were reconstructed with a standard delay and sum algorithm. A region of interest (ROI) was defined around the tube carrying the nanoshells, and the pixels within the ROI averaged to indicate the mean photoacoustic signal generation for the collected image (Figure S6c).

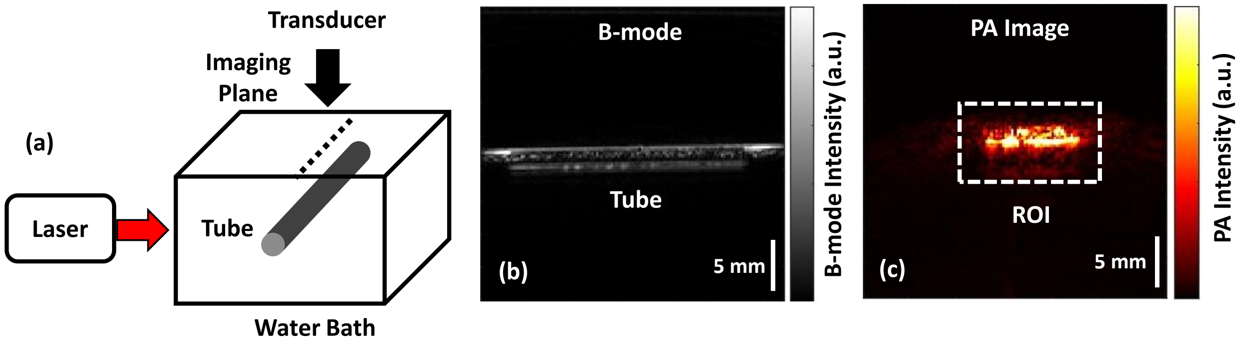

Figure S6. A description of the experimental phantom used for photoacoustic (PA) image acquisition. A tube carrying the Au nanoshells was placed under a water bath with optical illumination and acoustic detection, perpendicular to each other. Figures b) & c) describe the B-mode ultrasound and PA image collected from the tube. A region of interest is defined around the tube and the pixel intensities within this region were averaged to extract mean PA signals generated by a given nanoshell at exposed surface fluence. d) A plot of the average PA signal measured for

the 62 nm (black circles) and 102 nm (red circles) nanoshells at varying surface fluences. The error bars correspond to the variation in the average PA signal across five repeat acquisitions

a) Photo-stability of Contrast agents

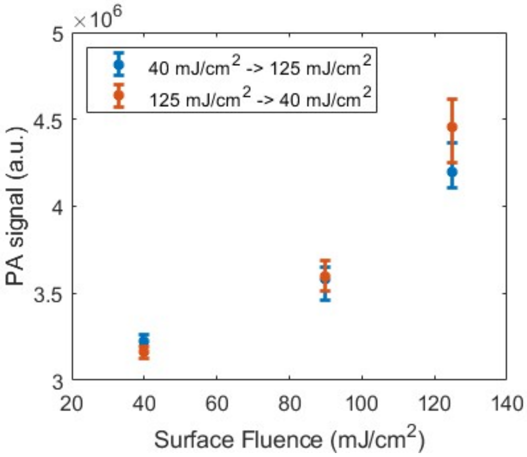

Figure S7: Verification of the thermal stability of the absorption dominant nanoshells under high fluences. The measured PA signal of the absorption dominant (62 nm) particles is thermally stable up to 120 mJ/cm^2. The surface exposure of the samples was increased from 40 to 120 mJ/cm^2 (blue circles) and back to 40 mJ/cm^2 (orange circles).

To demonstrate photostability of the contrast agents, PA signal was acquired from a sample of the absorption dominant nanoparticles, first increasing laser fluence from 40 to 125 mJ/cm^2, and then decreasing the fluence back to 40 mJ/cm^2 (Fig. S7). The characterization was performed without the preamplifier described above, reducing the dynamic range of the system to ~45 dB, since the PA signal within this fluence range saturates the signal from the preamplifier.

References

(1) Oldenburg, S. J.; Averitt, R. D.; Westcott, S. L.; Halas, N. J. Nanoengineering of Optical Resonances. *Chem. Phys. Lett.* **1998**, *288* (2), 243–247. https://doi.org/https://doi.org/10.1016/S0009-2614(98)00277-2.

(2) Duff, D. G.; Baiker, A.; Edwards, P. P. A New Hydrosol of Gold Clusters. 1. Formation and Particle Size Variation. *Langmuir* **1993**, *9* (9), 2301–2309.

(3) Steinhauser, G.; Evers, J.; Jakob, S.; Klapötke, T. M.; Oehlinger, G. A Review on Fulminating Gold (Knallgold). *Gold Bull.* **2008**, *41* (4), 305–317.

(4) Stöber, W.; Fink, A.; Bohn, E. Controlled Growth of Monodisperse Silica Spheres in the Micron Size Range. *J. Colloid Interface Sci.* **1968**, *26* (1), 62–69. https://doi.org/https://doi.org/10.1016/0021-9797(68)90272-5.

(5) Bogush, G. H.; Tracy, M. A.; Zukoski Iv, C. F. Preparation of Monodisperse Silica Particles: Control of Size and Mass Fraction. *J. Non. Cryst. Solids* **1988**, *104* (1), 95–106.

(6) Danielli, A.; Favazza, C. P.; Maslov, K.; Wang, L. V. Picosecond Absorption Relaxation Measured with Nanosecond Laser Photoacoustics. *Appl. Phys. Lett.* **2010**, *97* (16). https://doi.org/10.1063/1.3500820.

(7) Jain, P. K.; Lee, K. S.; El-Sayed, I. H.; El-Sayed, M. A. Calculated Absorption and Scattering Properties of Gold Nanoparticles of Different Size, Shape, and Composition: Applications in Biological Imaging and Biomedicine. *J. Phys. Chem. B* **2006**, *110* (14), 7238–7248. https://doi.org/10.1021/jp057170o.

(8) Wang, L. V; Wu, H. Rayleigh Theory and Mie Theory for a Single Scatterer. *Biomed. Opt. Princ. imaging, LV Wang* **2007**, 17–35.
